# Time trend and spatial distribution of the cases of lethal violence against women in Brazil

**DOI:** 10.1590/1518-8345.5613.3547

**Published:** 2022-07-15

**Authors:** Márcia Moroskoski, Franciele Aline Machado de Brito, Rosana Rosseto de Oliveira

**Affiliations:** 1 Universidade Estadual de Maringá, Maringá, PR, Brasil.; 2 Bolsista da Coordenação de Aperfeiçoamento de Pessoal de Nível Superior (CAPES), Brasil.

**Keywords:** Homicide, Gender-Based Violence, Violence Against Woman, Intimate Partner Violence, Epidemiology, Nursing, Homicídio, Violência de Gênero, Violência contra a Mulher, Violência por Parceiro Íntimo, Epidemiologia, Enfermagem, Homicidio, Violencia De Género, Violencia Contra La Mujer, Violencia de Pareja, Epidemiología, Enfermería

## Abstract

**Objective:**

to analyze the time trend and the spatial distribution of the cases of lethal violence against women in Brazil, according to age group and to race/skin color.

**Method:**

an ecological study of time series, with spatial distribution of the deaths of women victims of aggression, registered in the Mortality Information System, resident in Brazil, Brazilian geographic regions and states. Due to underreporting of deaths in some states, correction factors of the mortality rates were employed. For the trend analysis, we adopted the polynomial regression model. In addition to that, the mean rates and annual upward/downward trends were distributed considering the Brazilian federative units as analysis units.

**Results:**

the mean rate was 6.24 cases of lethal violence *per* 100,000 women, with a significant variation across the Brazilian regions and states. The main victims of violent death in Brazil are young, black-/brown-skinned and indigenous women, with a growing trend in these population segments. The North and Northeast regions stood out with the most significant mean annual increases (0.33; r^2^= 0.96 and 0.26; r^2^= 0.92, respectively).

**Conclusion:**

there was a stable trend regarding lethal violence against women, with significant regional differences. Young, black-/brown-skinned and indigenous women are more vulnerable to violent death in Brazil.

## Resumo

**Objetivo:**

analisar a tendência temporal e a distribuição espacial dos casos de violência letal contra mulheres no Brasil, segundo faixa etária e raça/cor.

**Método:**

estudo ecológico, de séries temporais, com distribuição espacial dos óbitos de mulheres vítimas de agressão, cadastrados no Sistema de Informação sobre Mortalidade, residentes no Brasil, regiões geográficas e estados brasileiros. Devido ao sub-registro de óbitos em alguns estados, empregaram-se fatores de correção das taxas de mortalidade. Para a análise de tendência, adotamos o modelo de regressão polinomial. Além disso, as taxas médias e as tendências de aumento/reduções anuais foram distribuídas considerando como unidade de análise as unidades federativas do Brasil.

**Resultados:**

a taxa média foi de 6,24 casos de violência letal por 100 mil mulheres, com variação importante entre as regiões e os estados brasileiros. As principais vítimas de morte violenta no Brasil são mulheres jovens, pretas/pardas e indígenas, com tendência de crescimento nessas populações. As Regiões Norte e Nordeste se destacaram com os aumentos anuais médios mais expressivos (0,33; r^2^ = 0,96 e 0,26; r^2^ = 0,92, respectivamente).

**Conclusão:**

evidenciou-se tendência de estabilidade da violência letal contra a mulher, com diferenças regionais significativas. Mulheres jovens, pretas/pardas e indígenas são mais vulneráveis à morte violenta no Brasil.

## Resumen

**Objetivo:**

analizar la tendencia temporal y la distribución espacial de los casos de violencia letal contra la mujer en Brasil, según la franja etaria y la raza/color.

**Método:**

estudio ecológico, de las series temporales, con distribución espacial de las muertes de mujeres víctimas de agresión, registradas en el Sistema de Información de Mortalidad, residentes en Brasil, regiones geográficas y estados brasileños. Debido a que hay subregistro de las muertes en algunos estados, se utilizaron factores de corrección para las tasas de mortalidad. Para el análisis de tendencias, adoptamos el modelo de regresión polinomial. Además, las tasas medias y las tendencias anuales de aumento/disminución fueron distribuidas considerando como unidad de análisis las unidades federativas de Brasil.

**Resultados:**

la tasa promedio fue de 6,24 casos de violencia letal por cada 100.000 mujeres, con variación significativa entre regiones y estados brasileños. Las principales víctimas de muerte violenta en Brasil son mujeres jóvenes, negras/morenas e indígenas y la tendencia es creciente en estas poblaciones. Las regiones Norte y Noreste presentaron los aumentos medios anuales más significativos (0,33; r^2^ = 0,96 y 0,26; r^2^ = 0,92, respectivamente).

**Conclusión:**

hubo una tendencia a la estabilidad de la violencia letal contra la mujer, con diferencias regionales significativas. Las mujeres jóvenes, negras/morenas e indígenas son más vulnerables a la muerte violenta en Brasil.

**Highlights:**

(1) There was an increase in the number of cases of lethal violence against women in Brazil, with regional disparities.

(2) The Brazilian North and Midwest regions recorded the highest mean rates.

(3) The North and Northeast regions had the most significant annual increase.

(4) Upward trend in the cases of lethal violence against young women (aged from 15 to 39 years old).

(5) Upward trend in the cases of lethal violence against black-/brown-skinned and indigenous women.

## Introduction

Gender violence is a global social and public health problem, whose final stage is femicide, a crime that has been responsible for victimizing thousands of women every year^1^.

In recent decades, discussions on how to define and measure femicide have increased significantly, driven by the high incidence in Latin America and the recent laws implemented in several countries that have harshened its punishment^2^.

Identification of the femicide cases is extremely relevant for the statistical analysis and implementation of effective public policies to combat violence against women. However, a number of researchers report certain difficulty sizing the femicide occurrences due to nonexistence of information sources that allow such identification^3^ ^-^ ^5^.

This scenario is aggravated in Brazil due to the Government’s slowness to investigate and process femicides, in addition to not providing information on closed cases. Due to the absence of concrete data, the research studies on the theme are carried out with data on female mortality due to aggression, currently considered as an indirect femicide indicator^6^.

In 2017, the United Nations (UN) estimated a global rate of 1.3 cases of lethal violence against women perpetrated by intimate partners or other family members *per* 100,000 women. Africa was the region where women presented the highest risk of being killed, recording a rate of 3.1 *per* 100,000 in the same year, followed by the Americas (1.6/100,000), Asia (0.9/100,000) and Europe (0.7/100,000)^7^.

In this segment, more than half of the countries with the highest femicide rates are in South America, Central America and the Caribbean. Murder involving couples and murder of women preceded by rape are the most common forms of the crime^8^ in Latin America.

In Brazil, the indicators of lethal violence against women draw the attention. In 2019, the rate was 3.5 cases *per* 100,000 women, a 17.3% reduction when compared to 2018, when 4.3 cases *per* 100,000 women were recorded. Although this result seems to be positive news, the analysis should be cautious because, in the same period, there was a 21.6% increase in violent deaths of women without indication of the cause, whether murder, accident or suicide^9^.

Among the main risk factors are gender inequality, previous domestic violence, family history of violence, low schooling, unemployment, poverty, young age, ethnicity and race/skin color, and access to weapons by the aggressor^10^ ^-^ ^11^.

Recently, community violence, alcohol and drug use, adverse childhood experiences, conditions of exacerbated disparity and family violence have been described as social comorbidities that increase the probability of femicide^12^.

In order to reduce the number of cases of violence against women, some international policies were instituted, such as the Sustainable Development Goals, which listed “achieving gender equality and empowering all women and girls” as one of its targets^13^.

The Brazilian policies for the protection of women also advanced with the publication of the Maria da Penha Law, in force since 2006, which devised mechanisms to curb domestic and family violence against women^14^, although experts point out that there was no reduction in the cases of domestic violence after implementing this law^15^.

In 2015, the Brazilian State also sanctioned the Femicide Law, which amended the penal code to provide for femicide as an aggravating circumstance for the crime of murder^16^.

Due to the high rates of lethal violence against women in Brazil and to the scarcity of studies on the theme, especially after entry into force of the Femicide Law, the objective of this research was to analyze the time trend and the spatial distribution of the cases of lethal violence against women in Brazil, according to age group and to race/skin color.

## Method

### Study design

This is an ecological study of time series, with spatial distribution of the rates of lethal violence against women, registered in the Mortality Information System (*Sistema de Informação sobre Mortalidade*, SIM).

### Study locus

The data on lethal violence against women living in Brazil, Brazilian geographic regions and states were analyzed.

Located in South America, Brazil has terrestrial borders with Uruguay, Argentina, Paraguay, Bolivia, Peru, Colombia, Venezuela, Guyana, Suriname and French Guiana, and it limits with the Atlantic Ocean. With an area of 8,510,295 km², it consists of five regions: North, Northeast, Midwest, Southeast and South; 27 federal units; one Federal District; and 5,570 municipalities. It had an estimated population of 212,559,409 inhabitants in 2020, at that time being the sixth most populous country in the world, with a *per capita* Gross Domestic Product (GDP) of R$ 35,161.70 in 2019^17^.

In 1976, Brazil began implementation of the SIM, with the objective of gathering mortality data from its population and, up to the present day, it has shown good performance^18^. Health Information Systems (HISs) are important tools for management, as they contribute to the quality of the services, reduce costs and enable research and teaching activities^19^.

### Population and data source

The population consisted of cases of lethal violence against women aged from 15 to 59 years old. This age group was defined after a previous analysis of the SIM database indicated a higher incidence in this age group. 

Women aged 60 years old or more were excluded because violence against older adults follows a different dynamic. A study evidenced that, in most cases, the aggressors of children/adolescents and adult women are the partners, while in aged women they are family members^20^. 

Among the age group selected for the analysis there was stratification from 15 to 39 years old, which in this study were considered young women, and from 40 to 59 years old, thus allowing a comparison of the rates between both age groups.

Data collection took place in March and April 2020 and in December 2021. 

The period analyzed was from 2000 to 2019 due to important laws having been implemented by the Brazilian State during those years, such as the compulsory notification of cases of violence in 2003, the Maria da Penha Law in 2006, and the Femicide Law in 2015^14^ ^,^ ^16^ ^,^ ^21^.

The indicators of lethal violence against women were selected in the SIM using codes X85 to Y09 of the International Classification of Diseases and Health-Related Problems (ICD-10). These codes refer to all forms of aggression, analyzed from the gender perspective because they are considered a proxy for femicide^6^.

The dependent variable was the rate of lethal violence against women. The other variables analyzed were related to age (from 15 to 39 years old and from 40 to 59 years old) and to race/skin color (White; Black/Brown, Asian and Indigenous). The race/skin color categories adopted in the study are in line with the Brazilian Institute of Geography and Statistics (*Instituto Brasileiro de Geografia e Estatística*, IBGE) and with the SIM.

### Data analysis

Calculation of the rates of lethal violence against women was performed year by year, and the ratio was determined by the number of cases of lethal violence against women and the female population living in the same place and period, multiplied by 100,000. The files with the mortality data and population estimates were extracted from the Informatics Department of the Unified Health System (DATASUS)^22^. 

The rates of lethal violence against women were corrected in order to point out the most precise incidence of the problem. The correction factors were developed through the project entitled “Active search for deaths and births in the Legal Amazon and in the Northeast”, which identified the proportion of live births and deaths not reported to the respective information systems^23^. These correction factors were multiplied by the rates of lethal violence against women, according to the Brazilian federative units and regions, in order to minimize SIM underreporting.

For the trend analysis, the polynomial regression model was used because of its high statistical power. Thus, the rates of lethal violence against women were considered as dependent variables (Y) and the years of study as independent variables (X). The “year” variable was converted into a centralized variable in year (X-2009) and the series were smoothed using a three-point moving mean^24^.

The polynomial regression models were tested as linear, considering the significant trend when the estimated model obtained *p*-value < 0.05. For the choice of the best model, the analysis of the dispersion plot, the value of the coefficient of determination (r² - the closer to 1, the more adjusted the model is) and the analysis of residuals (assumption of true homoscedasticity) were considered. When all the criteria were significant for more than one model and the coefficient of determination was similar, the simplest model was chosen^25^. The analyses were performed using the SPSS software, version 20.1.

For the spatial distribution, the cartographic base of Brazil with the borders of the states was downloaded in *shapefile*(SHP) format from the IBGE website.

Choropleth maps were prepared to show the spatial distribution of the mean rate across the federative units, as well as the mean annual upward/downward trends, according to race/skin color. For this stage, the analysis units were the Brazilian federative units and the Federal District. 

The spatial distribution of the rates of lethal violence against women was presented in intervals, from the maximum to the minimum rates, and the maps were represented on gray scales, defining lighter colors for lower rates and darker colors for higher rates. In turn, distribution of the mean annual increases or reductions was presented using gray for the states that recorded a reduction and black for those that presented an increase. All figures were prepared using the QGIS software, version 2.14.

### Ethical aspects

As this is a research study with data in the public domain, waiver of analysis was requested to the Permanent Committee of Ethics in Research Involving Human Beings of the State University of Maringá (*Comitê Permanente de* Ética *em Pesquisa Envolvendo Seres Humanos/Universidade Estadual de Maringá*, COPEP/UEM), obtaining approval through Edict No. 33/2019.

## Results

**Figure 1:**
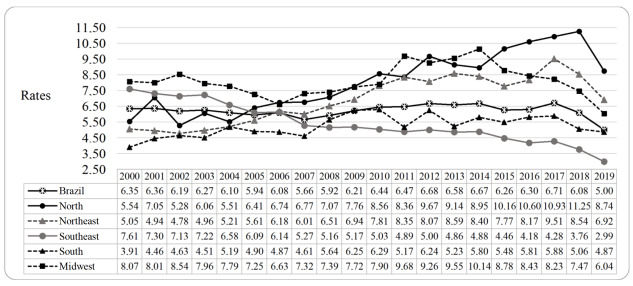
Rates of lethal violence against women (*per* 100,000 women), according to Brazilian regions. Brazil, 2000-2019

A total of 72,630 cases of lethal violence against women aged from 15 to 59 years old were analyzed in Brazil, from 2000 to 2019. In 2000, the national rate was 6.35, rising to 5.00*per* 100,000 women in 2019. In relation to the Brazilian regions, the North and Northeast stood out, rising from 5.54 and 5.05 in 2000 to 8.74 and 6.92 in 2019, respectively. In contrast, the Southeast presented a reduction from 7.61 in 2000 to 2.99 in 2019 (Figure 1)

**Figure 2:**
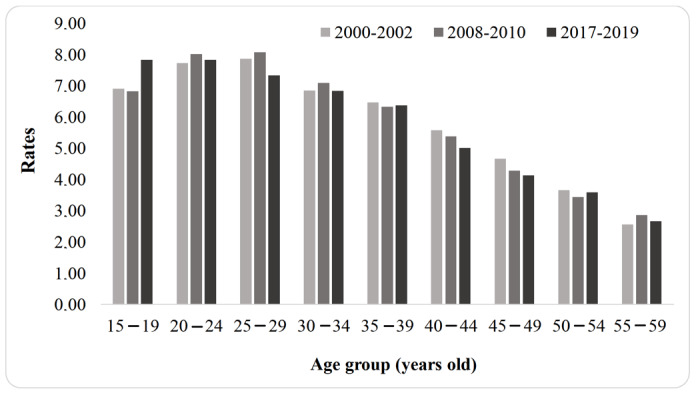
Lethal violence against women in Brazil (*per* 100,000 women), according to age groups. Brazil, 2000-2019

The cases of lethal violence against women in Brazil were grouped into three periods (2000-2002, 2008-2010 and 2017-2019), according to the age groups. The rates remained high throughout the period, although a reduction in the third three-year period (2017-2019) is observed in almost all age groups. Another important aspect concerns the significant reduction in the rates as the women’s age increases, evidencing that the main victims of lethal violence in Brazil are young women (Figure 2).

The polynomial regression analysis showed a stable trend regarding the rates of lethal violence against women in Brazil (0.02 *per* year). The Brazilian regions with the highest mean rates were Midwest (8.17) and North (7.89). The North region also stood out for the highest mean annual increase (0.33 *per* year; r^2^= 0.96).

In relation to the states of the North region, Rondônia recorded the highest mean rate (8.89), and Pará the highest mean annual increase (0.48; r^2^= 0.97). In the Northeast, the state of Alagoas had the highest mean rate (11.23), and Sergipe presented the highest mean annual increase among Brazilian states (0.63; r^2^= 0.92). In the Southeast, Espírito Santo stood out with the highest mean rate among the Brazilian states (13.47), while Minas Gerais had the highest mean annual increase (0.03; r^2^= 0.86). Also noteworthy is the downward trend in the rates in the states of São Paulo (-0.27 *per* year; r^2^= 0.87) and Rio de Janeiro (-0.21 *per* year; r^2^= 0.69).

In the South region, the state of Paraná presented the highest mean for the period (7.27) and Rio Grande do Sul, the highest mean annual increase (0.14; r^2^= 0.87). Finally, in the Midwest, Mato Grosso recorded the highest mean rate in the period (9.50) and Goiás, the highest mean annual increase (0.24; r^2^= 0.50) (Table 1).

**Table 1:** Trends in the rates of lethal violence against women (*per* 100,000 women). Brazil, 2000-2019

Locus	Mean rate	Mean annual Increase/Reduction	R^2^*	*p* ^†^	Trend
**Brazil**	Y=6.24	+0.02X	0.13	0.134	Stable
**North Region**	Y=7.89	+0.33X	0.96	<0.001	Upward
Acre	Y=7.79	+0.21X	0.64	<0.001	Upward
Amapá	Y=7.99	+0.00X	0.00	0.997	Stable
Amazonas	Y=6.64	+0.34X	0.93	<0.001	Upward
Pará	Y=8.07	+0.48X	0.97	<0.001	Upward
Rondônia	Y=8.89	+0.19X	0.70	<0.001	Downward/Upward/Downward
Roraima	Y=9.14	+0.21X	0.28	0.023	Upward
Tocantins	Y=7.28	+0.23X	0.52	0.001	Upward
**Northeast Region**	Y=6.86	+0.26X	0.92	<0.001	Upward
Alagoas	Y=11.23	+0.21X	0.83	<0.001	Upward/Downward
Bahia	Y=6.75	+0.41X	0.83	<0.001	Upward
Ceará	Y=6.61	+0.44X	0.84	<0.001	Upward
Maranhão	Y=5.17	+0.29X	0.80	<0.001	Upward
Paraíba	Y=8.03	+0.33X	0.75	<0.001	Upward/Downward
Pernambuco	Y=8.74	-0.16X	0.68	<0.001	Downward
Piauí	Y=3.91	+0.14X	0.71	<0.001	Upward
Rio Grande do Norte	Y=4.41	+0.37X	0.98	<0.001	Upward
Sergipe	Y=5.92	+0.63X	0.92	<0.001	Downward/Upward/Downward
**Southeast Region**	Y=5.50	-0.20X	0.94	<0.001	Downward
Espírito Santo	Y=13.47	-0.17X	0.84	<0.001	Upward/Downward
Minas Gerais	Y=6.38	+0.03X	0.86	<0.001	Upward/Downward
Rio de Janeiro	Y=6.43	-0.21X	0.69	<0.001	Downward
São Paulo	Y=4.46	-0.27X	0.87	<0.001	Downward
**South Region**	Y=5.58	+0.08X	0.80	<0.001	Upward/Downward
Paraná	Y=7.27	+0.04X	0.83	<0.001	Upward/Downward
Rio Grande do Sul	Y=5.19	+0.14X	0.87	<0.001	Upward
Santa Catarina	Y=3.61	+0.06X	0.84	<0.001	Upward
**Midwest Region**	Y=8.17	+0.06X	0.14	0.123	Stable
Federal District	Y=5.66	-0.02X	0.01	0.652	Stable
Goiás	Y=8.98	+0.24X	0.50	0.001	Upward
Mato Grosso	Y=9.50	-0.11X	0.40	0.005	Downward
Mato Grosso do Sul	Y=7.35	-0.08X	0.32	0.015	Downward

*R^2^= Coefficient of determination; ^†^ *p = p*-value, significant when < 0.05

With regard to age, there was an upward trend in the cases in young women in Brazil, aged from 15 to 39 years old (with a mean of 0.04 *per* year; r^2^= 0.36). Analyzing the regions, the Southeast presented a downward trend (-0.24 *per* year; r^2^= 0.95), as well as the South region, in the final analysis period. On the other hand, the other regions showed an upward trend, with the highest annual increase in the North (0.39 *per* year; r^2^= 0.95). The highest mean rate was recorded in the Midwest (9.34/100,000).

In relation to the Brazilian states, Espírito Santo recorded the highest mean rate of the period in women aged from 15 to 39 years old: 16.39 *per* 100,000 women, followed by Alagoas: 12.93 *per* 100,000. The most significant mean annual increases were in Ceará (0.58; r^2^= 0.81) and Pará (0.57; r^2^= 0.97).

The polynomial regression analysis also showed an increase in lethal violence against women in Brazil in the age group from 40 to 59 years old (0.04; r^2^= 0.49), with an initial and final period characterized by a reduction. The North, Northeast and South regions follow an upward trend, with the highest mean annual increase in the North (0.20; r^2^= 0.94). A downward trend was recorded in the Southeast (-0.10; r^2^= 0.89), while there was stability in the rates in the Midwest (*p*= 0.591).

In this same age group, the highest mean rates among the Brazilian states were seen in Roraima (8.79) and Espírito Santo (7.81). The highest annual increases were recorded in Pará (0.30; r^2^= 0.92) and Rio Grande do Norte (0.26; r^2^= 0.96). Finally, it is worth noting the significant annual reduction in Acre (-0.34 *per* year; r^2^= 0.61) (Table 2).

**Table 2:** Trends in the rates of lethal violence against women (*per* 100,000 women) according to the age group. Brazil, 2000-2019

Locus	15-39 years old					40-59 years old				
	Mean rate	Mean annual Increase/ Reduction	R^2^*	*p* ^†^	Trend	Mean rate	Mean annual Increase/ Reduction	R^2^*	*p* ^†^	Trend
**Brazil**	Y=7.32	+0.04X	0.36	0.009	Upward	Y=4.19	+0.04X	0.49	0.006	Downward/Upward/Downward
**North Region**	Y=8.67	+0.39X	0.95	<0.001	Upward	Y=5.91	+0.20X	0.94	<0.001	Upward
Acre	Y=8.31	+0.28X	0.63	<0.001	Upward	Y=7.22	-0.34X	0.61	0.001	Upward/Downward/Upward
Amapá	Y=8.49	-0.02X	0.05	0.791	Stable	Y=6.42	+0.11X	0.07	0.0275	Stable
Amazonas	Y=7.23	+0.42X	0.95	<0.001	Upward	Y=5.09	+0.14X	0.47	0.002	Upward
Pará	Y=8.93	+0.57X	0.97	<0.001	Upward	Y=5.93	+0.30X	0.92	<0.001	Upward
Rondônia	Y=9.81	+0.26X	0.69	<0.001	Downward/Upward/Downward	Y=7.35	+0.06X	0.12	0.156	Stable
Roraima	Y=10.03	+0.25X	0.17	0.092	Stable	Y=8.79	+0.28X	0.65	<0.001	Upward/Downward
Tocantins	Y=8.12	+0.31X	0.60	<0.001	Upward	Y=5.33	+0.09X	0.15	0.106	Stable
**Northeast Region**	Y=7.94	+0.34X	0.93	<0.001	Upward	Y=4.64	+0.13X	0.91	<0.001	Upward
Alagoas	Y=12.93	+0.26X	0.84	<0.001	Upward/Downward	Y=7.50	+0.13X-0.04X^2^	0.55	0.001	Upward/Downward
Bahia	Y=7.94	+0.51X	0.84	<0.001	Upward	Y=4.35	+0.25X	0.87	<0.001	Upward
Ceará	Y=7.57	+0.58X	0.81	<0.001	Upward	Y=4.72	+0.20X	0.92	<0.001	Upward
Maranhão	Y=5.66	+0.32X	0.81	<0.001	Upward	Y=3.96	+0.25X	0.65	<0.001	Upward
Paraíba	Y=9.83	+0.46X	0.67	<0.001	Upward/Downward	Y=3.90	+0.10X	0.36	0.008	Upward
Pernambuco	Y=10.31	-0.11X	0.41	0.004	Downward	Y=5.65	-0.19X	0.81	<0.001	Downward
Piauí	Y=4.30	+0.15X	0.56	<0.001	Upward	Y=3.09	+0.16X	0.82	<0.001	Upward
Rio Grande do Norte	Y=7.35	+0.67X	0.97	<0.001	Upward	Y=3.84	+0.26X	0.96	<0.001	Upward
Sergipe	Y=7.01	+0.31X	0.67	<0.001	Upward	Y=4.69	+0.11X	0.21	0.055	Stable
**Southeast Region**	Y=6.56	-0.24X	0.95	<0.001	Downward	Y=3.68	-0.10X	0.89	<0.001	Downward
Espírito Santo	Y=16.39	-0.13X	0.82	<0.001	Upward/Downward	Y=7.81	-0.19X	0.75	<0.001	Downward
Minas Gerais	Y=7.84	+0.05X	0.88	<0.001	Upward/Downward	Y=3.86	+0.04X	0.71	<0.001	Upward/Downward
Rio de Janeiro	Y=7.78	-0.26X	0.71	<0.001	Downward	Y=4.30	-0.11X	0.49	0.001	Downward
São Paulo	Y=5.22	-0.34X	0.87	<0.001	Downward	Y=3.09	-0.13X	0.88	0.001	Downward
**South Region**	Y=6.80	+0.12X	0.82	<0.001	Upward/Downward	Y=3.68	+0.04X	0.59	<0.001	Upward
Paraná	Y=8.85	+0.06X	0.82	<0.001	Upward/Downward	Y=4.62	+0.05X	0.59	0.001	Upward/Downward
Rio Grande do Sul	Y=6.18	+0.21X	0.90	<0.001	Upward	Y=3.74	+0.05X	0.54	0.001	Upward
Santa Catarina	Y=4.17	+0.09X	0.78	<0.001	Upward	Y=2.66	+0.02X	0.12	0.163	Stable
**Midwest Region**	Y=9.34	+0.11X	0.25	0.036	Upward	Y=5.83	+0.01X	0.02	0.591	Stable
Federal District	Y=7.42	+0.21X	0.60	0.001	Downward/Upward/Downward	Y=3.54	+0.06X	0.20	0.062	Stable
Goiás	Y=10.48	+0.36X	0.55	<0.001	Upward	Y=6.11	+0.09X	0.43	0.003	Upward/Downward
Mato Grosso	Y=10.29	-0.08X	0.24	0.039	Downward	Y=7.15	-0.17X	0.62	<0.001	Upward/Downward
Mato Grosso do Sul	Y=8.38	-0.08X	0.27	0.028	Downward	Y=5.38	-0.05X	0.07	0.292	Stable

*R^2^= Coefficient of determination; ^†^ *p = p*-value, significant when < 0.05

With regard to race/skin color, through the spatial distribution of the rates, it is evident that the main victims of lethal violence in Brazil are black- and brown-skinned women, mainly concentrated in the North, Northeast and Midwest regions of the country. The states with the highest rates in this population segment were Espírito Santo (9.32), Alagoas (8.79) and Pernambuco (7.04). It is also noted that most of the states presented a significant mean increase in the number of cases in black- and brown-skinned women, especially Pará (0.48*per* year; r² = 0.97), Rio Grande do Norte (0.45 *per* year; r² = 0.96), Bahia (0.39 *per* year; r² = 0.85), Ceará (0.44 *per* year; r² = 0.75) and Alagoas (0.36 *per* year; r² = 0.80).

The highest rates in white-skinned women were recorded in the Brazilian South, Midwest and part of the Southeast region. The state of Paraná stood out with the highest mean rate among white-skinned women (5.70), followed by Rio Grande do Sul (4.24). A mean annual reduction is also noticed in the number of cases in white-skinned women in most states, with emphasis on São Paulo (-0.18; r² = 0.91), Rio de Janeiro (-0.13; r² = 0.78) and Mato Grosso (-0.14; r² = 0.89). 

Lethality against Asian-descendant women was more evenly distributed across the Brazilian regions. However, the states of Amapá (0.15), Piauí (0.037), Tocantins (0.023), São Paulo (0.023) and Mato Grosso (0.022) presented the highest rates. In turn, the states of Acre and Roraima presented the highest mean annual increases (0.009; r² = 0.34 and 0.009; r² = 0.17).

**Figure 3:**
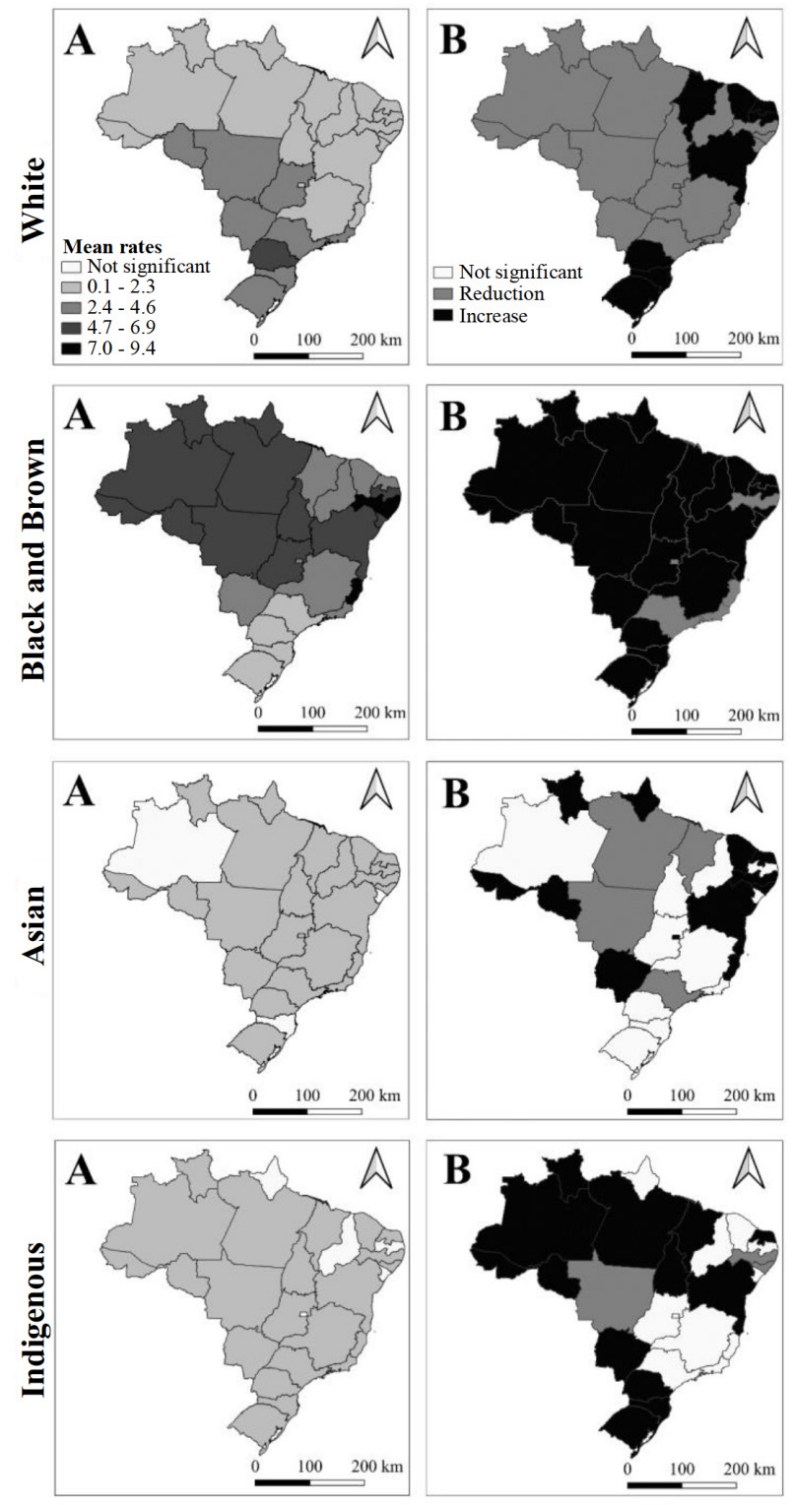
Spatial distribution of the mean rates and mean annual increase/reduction in lethal violence against women (*per* 100,000 women) in the Brazilian states, according to race/skin color. Brazil, 2000-2019

Finally, violent deaths of indigenous women were recorded in most of the states of the country. Roraima presented the highest mean rate (1.13) and the highest mean annual increase (0.12 *per* year; r² = 0.38), followed by Mato Grosso do Sul (0.70), with a mean increase of 0.03 *per* year (r² = 0.41) (Figure 3).

A = Spatial distribution of the mean rates of lethal violence against women, by race/skin color. Brazil (2000-2019); B = Spatial distribution of the mean annual increase/reduction in lethal violence against women, by race/skin color

## Discussion

There is still scarcity of time trend and spatial distribution analyses of the rates of lethal violence against women that consider the regional differences in Brazil, age group and race/skin color. This research pointed to a stable trend regarding lethal violence in Brazil, with important variations across the five regions of the country and the Brazilian states. The results indicate that, without the implementation of effective actions, the cases of violent death among young, black-/brown-skinned and indigenous women will continue to grow.

This study has limitations in relation to the use of secondary data, considered sensitive sources, due to incompleteness of information and underreporting of deaths. In this sense, in order to circumvent under-recording, especially in some less favored regions of the country, the research implemented correction factors for the rates, contributing to a more precise analysis. In addition, the SIM has expanded its coverage and shown good performance^18^, being fundamental for conducting ecological studies on the mortality of the Brazilian population in the national scope. 

In addition to that, for the spatial analysis, the data were aggregated in time and space. Therefore, new studies may be carried out disaggregating the data in relation to the period and expanding the analysis units. 

Another limitation concerns the nonexistence of a national database with specific femicide cases, causing the study to investigate lethal violence against women from a gender perspective. However, it is noteworthy that the analysis contributed to identifying the differences across the Brazilian territories, enabling targeting of actions to control the problem. 

The debates on femicide, a crime that involves gender-motivated lethal violence against women, have gained space in recent decades influenced by the high incidence in Latin America^2^.

The increase in the number of cases exerted pressure on several countries’ governments to implement specific laws that criminalize the murder of women on gender-related grounds, although the result of the legislation is not known due to the absence of a national database^26^ ^-^ ^27^. Currently, all Latin American countries except Cuba and Haiti have passed specific laws or implemented devices to fight against gender violence^28^.

In Brazil, the Maria da Penha Law, enacted in 2006, devised mechanisms to curb domestic and family violence against women^14^. Despite the significant progress, it is important to highlight that this law was the result of a condemnation of the Brazilian State by the Inter-American Court of Human Rights. On that occasion, the international sentence disclosed the inefficiency of the Brazilian State in duly prosecuting and holding the then husband of Maria da Penha Maia Fernandes, a Brazilian woman from Ceará, responsible after years of aggressions and two murder attempts^29^.

Some studies indicate that the law in question contributed to conferring visibility to the topic and encouraging women to report their aggressors, although the statistics do not indicate significant reductions in the number of cases after its implementation^30^.

In line with the literature, the data found in this research do not evidence a significant impact of the Maria da Penha Law on the rates of violent death of women in Brazil, given that in 2007 and 2008 there was a slight reduction with a subsequent sustained increase.

Another important aspect concerns the law’s weaknesses, as countless cases of new aggressions against women are recorded even after filing reports, showing the ineffectiveness of security institutions in ensuring the protection of women in situations of violence^31^.

Faced with this scenario of inequalities, a Joint Parliamentary Inquiry Commission (*Comissão Parlamentar Mista de Inquérito*, CPMI) was organized in 2013 in order to know the number of women victims of violent deaths in Brazil. It was evidenced that most of the cases were cruel deaths caused by intimate partners, a situation similar to the one recorded in Peru^30^ ^,^ ^32^.

This information, as well as the pressure exerted by the UN to confront violence against women worldwide^33^, contributed to the publication of Law 13,104/2015, known as the Femicide Law.

The aforementioned law created an aggravating circumstance for the crime of murder when committed against women for reasons of female status. Therefore, this aggravating circumstance should be applied when it involves “domestic and family violence” and also “contempt or discrimination to the woman’s condition”^16^.

The current study shows that, in 2016 and 2017, years after publication of the Femicide Law, the rates of lethal violence against women in Brazil continued to rise, followed by a reduction in the last two years of analysis (2018 and 2019), suggesting a possible positive effect of the legislation. 

However, further studies will be necessary to analyze the long-term effect, as well as the influence of the COVID-19 pandemic on the rates, as some studies already indicate an increase in the rates during the pandemic period in other Latin American countries, such as Colombia and Mexico^34^.

Globally, the rates of violent death of women follow divergent paths. While some countries have been recording an increase, such as Turkey, Peru and Ecuador, others such as Italy and Spain record a reduction in the numbers^35^ ^-^ ^39^. 

As observed in the world, these variations were seen across the Brazilian regions, where two of them stood out with the most significant mean annual increase: North and Northeast. The Midwest region presented a stable trend in the rates, while the Southeast and South regions (from 2010) recorded a downward trend, similarly to another Brazilian study^40^.

In general, the most expressive mean rates coincide with the socio-economically disadvantaged regions of the country: North and Northeast. On the other hand, the stable trend in the Midwest region and the reduction trend in the Southeast and South are consistent with the existence of better indicators^41^.

Other studies indicate a mean rate of 4.7 violent deaths *per* 100,000 women in the Northeast, while incidence was lower in Rio Grande do Sul: 3.2 *per* 100,000 women^42^ ^-^ ^43^. These data confirm the variability in the rates of lethal violence against women, a consequence of the disparities experienced and of the weaknesses of the public security services. 

As Brazil is a country with continental dimensions, the differences in the indicators across the regions result from social inequality, such as schooling level, unemployment, structural violence, race/skin color and ethnicity, in addition to territories under a strong influence of the traditional gender roles and religious norms, a reality that is similar to those of other Latin American countries^34^ ^,^ ^44^.

In turn, Brazil, Mexico and Colombia also recorded variations in the rates in their territories, associated with places with a higher concentration of poverty, lower schooling and locations with a high proportion of unmet basic needs and high rates of pregnancy during adolescence^45^ ^-^ ^46^.

In Spain, a research study evidenced that economic precariousness and unemployment trigger situations of violence against women, due to the financial dependence generated^47^. This allows us to infer that women who are economically dependent on their partners tend to remain in abusive and violent relationships, increasing the risk of femicide.

Brazilian inequality is also seen in the provision of protective devices for women. Data from the National Council of Justice (*Conselho Nacional de Justiça*, CNJ) show a disproportionate distribution of Judicial Units with exclusive competence for domestic and family violence against women across the Brazilian regions. 

In 2020, there were 138 exclusive courts, of which 34 were located in the Northeast, 16 in the North, 32 in the Midwest, 43 in the Southeast and 13 in the South. Centralization of the services is more evident when analyzing data from the federative units. Although the estimated female population in Amazonas was 2,095,586 in 2020, the state has only three exclusive courts. In contrast, the Federal District has 16 exclusive courts for a female population estimated at 1,587,124^48^. 

In line with these data, the number of Specialized Police Stations for Women (*Delegacias Especializadas em Atendimento à Mulher*, DEAMs) differs across the states. A number of studies indicate that Santa Catarina has 30 DEAMs for 3,654,387 women, while Maranhão has 19 specialized police stations for 3,618,170 women^49^ ^-^ ^50^. Thus, improving the socioeconomic conditions, as well as equal supply of protective devices for women, is fundamental for a change in the scenario of violence. 

In relation to the age group, all the Brazilian regions and states had a higher incidence of lethal violence against young women, aged from 15 to 39 years old. Similar data to those of the current study were reported in other surveys conducted in Brazil in which the age groups with the highest occurrence values were those from 15 to 34 years old^43^ and from 20 to 39 years old^44^.

Violent death of young women is a reality in other countries, such as Turkey, the United States, Ecuador and a region of South Africa^35^ ^,^ ^51^ ^-^ ^53^. These findings are alarming because femicide represents the final stage of a cycle of violence^54^ that shortens girls’ and women’s lives, leaving inestimable consequences for the families and society.

A number of studies indicate that the risk factors for femicide in adolescents and young women include relationship breakups, jealousy, intimate partner in possession of a firearm and suspected infidelity, as well as a history of stalking by the partner^55^ ^-^ ^57^.

Recently, the persecution habit, a behavior called stalking, has gained ground in the debates, and is even criminalized in some countries. In this logic, a research study conducted with adolescents in the United States evidenced that 51.1% of the girls had already being victims of stalking or harassment^58^.

Regarding race/skin color, higher rates of lethal violence were found among black- and brown-skinned women, concentrated in the Brazilian North, Northeast, Midwest and part of the Southeast region. The high rates in this population segment are consistent with the results of another study^59^. It is also noted that, while lethal violence in white-skinned women is decreasing in most states, it is increasing among black- and brown-skinned women, reasserting data from the literature^60^.

The high incidence in black- and brown-skinned women is a reflection of the situation of social vulnerability and disparity they experience, especially in the educational area, in the labor market, in income and in political participation^61^ ^-^ ^62^.

Such being the case, ethnic and social inequalities across the Brazilian regions lead us to reflect on the public policies, considering whether they have the same reach for the entire population. Identification of the women most affected by this problem reinforces the need to provide special and personalized assistance on the part of the women protection services, in articulation with the health services, the gateway to the care to be provided to victims of aggressions.

Likewise, an increase in lethal violence against indigenous women was recorded in several Brazilian states, a situation similar to that of the United States, where the highest rates correspond to black-skinned and indigenous women^51^.

In the last IBGE census (2010), Brazil had an estimated indigenous population of 817,962 inhabitants, of which 502,783 lived in rural areas and 315,180 in urban areas. Currently, the country has 488 regularized indigenous lands, which occupy approximately 12.2% of the national territory, distributed in the North (54%), Northeast (11%), Southeast (6%), South (10%) and Midwest (19%) regions^63^.

Lethal violence against indigenous women is perpetrated by indigenous or non-indigenous individuals. In the first case, the crime can be related to the consumption of alcohol and drugs. In the second, it is preceded by sexual violence in most of the cases. But there are also cases arising from leadership actions that confront the gender relations of their cultures^64^.

A number of researchers also draw the attention to the genocide that has been taking place in the dispute for territories, in which women from certain ethnic and race-related groups are killed as a message of terror^59^.

Another factor related to this increase is the interference of white-skinned people in indigenous communities. Situations such as the modification of the natural landscape and the introduction of alcohol and drugs contribute to making gender relations conflictive^65^. In addition, the migration of indigenous people to urban centers, living in a situation of social vulnerability in most of the cases, favors the increase in violence^66^. 

The UN highlights that institutional racism is an obstacle for black, brown and ethnic minority women to enjoy access to justice, as well as it aggravates the situation of vulnerability they experience^67^.

The World Health Organization (WHO) reiterates that violence against women is preventable and derives from social, cultural and economic factors, among others, being reduced through the implementation of effective programs and policies. In this sense, it becomes fundamental to analyze the socioeconomic level, as well as the existence of specific laws, income levels, poverty and economic deprivation, employment, schooling, access to health services and political participation^68^.

Concrete measures to support women in situations of violence prevent the occurrence of fatal crimes since, in most of the cases, death is preceded by episodes of psychological, patrimonial, physical and sexual violence^60^.

Finally, in the Health and Nursing context, knowing the regions with the highest incidence of lethal violence against women, as well as the predominant age group and race/skin color, favors the planning of comprehensive care, which aims at interrupting the cycle of violence, avoiding recurrence of fatal cases.

## Conclusion

There was a stable trend in the rates of lethal violence against women in Brazil, with important regional disparities. The North and Midwest regions recorded the highest mean rates, the North and Northeast regions had the most significant annual increase, and the Southeast was the only region that presented a significant reduction in the rates. 

An upward trend in the number of cases was evidenced in young women, in the age group from 15 to 39 years old. The spatial distribution of the rates, according to race/skin color, revealed higher incidence values in black- and brown-skinned women, with an upward trend. 

It is necessary to recognize the regional differences in Brazil so that the strategic planning of actions is based on the peculiarities of each location and on the risk and protection factors regarding violence against women.
